# Revealing the clinical phenotype of atypical neuronal ceroid lipofuscinosis type 2 disease: Insights from the largest cohort in the world

**DOI:** 10.1111/jpc.15250

**Published:** 2020-12-30

**Authors:** Charles M Lourenço, Andre Pessoa, Carmen C Mendes, Carolina Rivera‐Nieto, Diane Vergara, Mónica Troncoso, Emily Gardner, Francisca Mallorens, Lina Tavera, Luis A Lizcano, Nora Atanacio, Norberto Guelbert, Norma Specola, Nury Mancilla, Carolina F M de Souza, Sara E Mole

**Affiliations:** ^1^ School of Medicine Estácio University Center Ribeirão Preto São Paulo Brazil; ^2^ Pediatric Neurology Service, Albert Sabin Children's Hospital University of Ceará State Fortaleza Ceará Brazil; ^3^ Reference Center in Inborn Errors of Metabolism, Department of Pediatrics Universidade Federal de São Paulo São Paulo Brazil; ^4^ Medical Genetic Service Fundación Cardioinfantil Bogotá Colombia; ^5^ Service of Children Neuropsychiatry, San Borja Arriarán Hospital School of Medicine of the University of Chile Santiago Chile; ^6^ UCL MRC Laboratory for Molecular Cell Biology University College London London United Kingdom; ^7^ Medical Genetics Section Hospital Nacional Prof. A. Posadas Buenos Aires Argentina; ^8^ Foundation Neuroconexion Armenia Colombia; ^9^ Human Genetic Service Bogotá Colombia; ^10^ Dr. N.A Chamoles Laboratory Pedro de Elizalde Children's Hospital Buenos Aires Argentina; ^11^ Metabolic Disease Section Corboda Children's Hospital Buenos Aires Argentina; ^12^ Metabolic Unit Children Hospital of La Plata Buenos Aires Argentina; ^13^ Department of Paediatrics National University of Colombia Bogotá Colombia; ^14^ Medical Genetic Service Porto Alegre Clinic Hospital Porto Alegre Brazil; ^15^ UCL MRC Laboratory for Molecular Cell Biology and UCL Great Ormond Street Institute of Child Health University College London London United Kingdom

**Keywords:** Batten disease, late onset, mutation, seizure, TPP1 deficiency

## Abstract

**Aim:**

Neuronal ceroid lipofuscinosis type 2 (CLN2) disease is an autosomal recessive inherited neurodegenerative lysosomal storage disorder caused by deficient tripeptidyl peptidase 1 (TPP1) enzyme, leading to progressive deterioration of neurological functions commonly occurring in children aged 2–4 years and culminating in early death. Atypical cases associated with earlier or later symptom onset, or even protracted course, have already been reported. Such variable manifestations may constitute an additional challenge to early diagnosis and initiation of appropriate treatment. The present work aimed to analyse clinical data from a cohort of Latin American CLN2 patients with atypical phenotypes.

**Methods:**

Experts in inborn errors of metabolism from Latin America selected patients from their centres who were deemed by the clinicians to have atypical forms of CLN2, according to the current literature on this topic and their practical experience. Clinical and genetic data from the medical records were retrospectively revised. All cases were presented and analysed by these experts at an Advisory Board Meeting in São Paulo, Brazil, in October 2018.

**Results:**

Seizures, language abnormalities and behavioural disorders were found as the first manifestations, appearing at the median age of 6 years, an older age than classically described for the late infantile form. Three novel mutations were also identified.

**Conclusion:**

Our findings reinforce the inclusion of CLN2 in the differential diagnosis of children presenting with seizures, behavioural disorders and language abnormalities. Early diagnosis will allow early initiation of specific therapy.

## What is already known on this topic

1. Neuronal ceroid lipofuscinosis type 2 (CLN2) is an autosomal recessive neurodegenerative lysosomal storage disorder caused by deficient activity of the tripeptidyl peptidase 1 enzyme (TPP1).
2. The classic form (or late infantile), in which the onset of symptoms frequently occurs nearly 2–4 years, is the most well‐documented phenotype. This form includes language difficulties, seizures, sleep disturbances, ataxia, movement disorders, motor deterioration, dementia and visual loss, culminating with precocious death.

## What this paper adds

1. In this cohort, patients with atypical CLN2 disease presented with seizures, language abnormalities and behavioural disorders as first symptoms.
2. The median age of symptoms onset was 6 years, an older age than classically described for the late infantile form of this disease.
3. Three novel mutations were identified in these patients.

Neuronal ceroid lipofuscinosis type 2 (CLN2) is an autosomal recessive neurodegenerative lysosomal storage disorder caused by deficient activity of the tripeptidyl peptidase 1 enzyme (TPP1). This disease is part of a group of progressive neurogenetic disorders, the neuronal ceroid lipofuscinoses (NCLs), also known as Batten disease.^1^ Typically, pathogenic mutations in the *TPP1* gene result in the classical late infantile phenotype, with symptom onset between 2 and 4 years of age, including seizure, progressive neurodegeneration, visual failure and motor impairment.^2^ Variant forms with a later onset and a more protracted course have been reported world‐wide in a restricted number of patients.^2^, ^3^

The presentation of the classic disease form is generally considered to be consistent and uniform, being well defined by age of onset (late infantile) and multiple neurological signs and symptoms, as well as specific electrophysiological findings and pathological changes in magnetic resonance images.^4^ The clinical course includes language difficulties, seizures, sleep disorders, ataxia, movement disorders, motor deterioration, dementia, visual loss at the late stage of the disease and early death.^5^ Data from a database of 74 patients with late infantile CLN2 showed that the most common first symptoms are seizures (70%), language difficulty (57%), motor difficulty (41%), behavioural disorders (16%) and dementia (9%) and the median time between first symptoms and death is 7–8 years.^6^ Electroencephalograms may detect irregular activity, a slowing of background activity and epileptiform abnormalities in posterior regions. Findings of magnetic resonance images (MRI) may include progressive cerebellar and cerebral atrophy, reduction in grey matter volume and periventricular white matter hyperintensities. Mucosal biopsies to assess the accumulation of intracellular storage material typically reveal curvilinear profiles,^7^ which are a morphological hallmark of this particular NCL disorder. Mixed patterns of both curvilinear and fingerprint profiles may be associated with atypical CLN2 phenotypes.^4^ The definitive diagnosis is based on the determination of deficient TPP1 enzyme activity (in leukocytes, fibroblasts or dried blood spots (DBS)) and in the presence of pathogenic mutations in both alleles of the *TPP1* gene. Either demonstration of deficient TPP1 enzyme activity in leukocytes or fibroblasts, or detection of two pathogenic mutations *in trans* are considered diagnostic for CLN2 disease.^4^ At the moment, there are at least 139 mutations described in the current online NCL‐disease database mutation table.^8^

There are no cures for the NCLs yet. Many therapeutic attempts have been proposed, including enzyme supplementation, gene therapy, stem cell therapy, anti‐inflammatories and small molecules – but most of these are currently in early stages of clinical development.^9^ However, cerliponase alfa (Brineura), a recombinant human TPP1, is the first and recently approved therapy in USA, EU (EMA‐linked European countries including the UK), Ukraine, Brazil, Australia, Mexico, Canada, Colombia and Japan to treat the classic form of CLN2 disease.^10^ It has been demonstrated that regular intraventricular delivery of this enzyme may slow down the decline in motor and language function of classic CLN2 patients.^11^

Patients diagnosed with CLN2 disease may have a disease course that deviates from the typical clinical pattern. This atypical form is variable and broad in terms of age of onset, course of disease evolution, and multiple phenotypes, suggesting that there is a spectrum for this disease in terms of symptomatic presentation. Some of the atypical cases exhibit significant parkinsonian features, later age of onset, slower progression, and, remarkably, absence of seizures and visual loss.^12^ Other atypical cases with a demonstrated partial TPP1 enzyme deficiency show prominent ataxia, but absence of seizures, cognitive regression or visual findings.^13^, ^14^ Some of these cases were originally diagnosed with autosomal recessive spinocerebellar ataxia (type 7; SCAR7), besides low TPP1 enzymatic activity, making SCAR7 a possible differential diagnosis of CLN2 atypical cases.^14^

A recent survey of 25 CLN2 cases in South America showed that patients with a more severe phenotype (the classical form) had null TPP1 activity, while the group of patients with residual TPP1 activity showed later onset and longer life‐span, with slower progression, less epilepsy syndrome and a higher frequency of ataxia particularly at a late stage of the disease.^2^ These patients with residual enzyme activity are quite similar to many atypical cases described in the literature. The biological explanation for the variable disease presentation is not understood but is likely to include different levels of TPP1 deficiency arising from the type of mutation, a differential sensitivity of neuronal populations to TPP1 deficiency^15^ and/or other unknown molecular mechanisms.

Although the existence of atypical forms of CLN2 disease is recognised by experts in the field, description of the natural course of this form of the disease, especially in Latin America, remains overlooked. Therefore, the present study was tailored to collect data from patients with atypical CLN2 disease in Latin America, attempting to identify potential patterns of clinical presentation, enzymatic activity and/or genetic findings that could assist physicians anywhere to reach a diagnostic suspicion of CLN2 more effectively.

## Methods

A group of 13 Latin American experts in the field of inborn errors of metabolism were invited to participate in an Advisory Board meeting supported by BioMarin in São Paulo, Brazil, in October 2018. Before the meeting, these experts provided medical data from patients with atypical forms of CLN2 in their centres using a spreadsheet with multiple clinical variables such as first symptoms, age at disease onset, enzyme activity, diagnostic methods and genetic profiling. Data were presented and analysed by these experts throughout the Advisory Board meeting.

Inclusion criteria consisted of presenting with the atypical phenotype of CLN2. For that, an atypical phenotype is represented by earlier or later symptom onset, varied symptoms and/or slower progression but the maintenance of neurodegeneration and possible premature death.^2^, ^3^, ^12^, ^14^, ^15^ Exclusion criteria consisted of presentation with other phenotypes of CNL2. There was no sample size calculation because of the rarity of the condition. All patients were subjected to the enzymatic activity assay in DBS, leukocytes or saliva. Gene sequencing, exome evaluation and skin biopsy were also conducted in some patients.

After the meeting, the spreadsheets were revised to collect additional data and statistical analysis was performed. Descriptive statistics was used to perform the analysis, using frequencies and percentages to represent categorical data variables. Continuous data variables were summarised by the number of subjects (*n*), mean, standard deviation, median, minimum and maximum values.

### Declaration of patient consent

The authors certify that they have obtained all appropriate patient consent forms. In these forms, the patient's parents have given their consent for their children's images and all clinical information to be reported in the manuscript. The parents understood that the patients' names and initials would not be published, and that all efforts would be made to conceal their identity, but anonymity could not be guaranteed.

## Results

The cohort included 30 patients from South America with an atypical form of CLN2 disease, as shown in Table 1. The median age at symptom onset was 6 years (n = 29, MIN = 1, MAX = 41). The reported first symptoms of these patients were: seizures (47%, 14/30), language/speech alterations (20%, 6/30, which included reports of speech delay −2/30; language delay −1/30; language regression – 1/30 and language difficulties −2/30), behavioural abnormalities (7%, 2/30), cognitive impairment (7%, 2/30), visual alterations (3%, 1/30) and visual hallucination (3%, 1/30).

**Table 1:** Characterisation of patients with atypical CLN2 in our cohort

#Patient	TPP‐1 enzymatic activity, samples (NR)	Allele 1	Allele 2	Tissue electron microscopy	First clinical symptom	Age at first clinical symptom, years	Other symptoms that appeared at the same age of the 1st symptom	Affects siblings	Age of seizures onset, years	Age of ataxia onset, years	Age of myoclonus onset, years	Age of pyramidal signs onset, years	Age of cognitive decline onset, years	Age of language difficulties onset, years	Age of swallowing difficulty onset, years	Age of visual loss onset, years	Age of behavioural abnormality onset, years	Age of eye movement abnormalities onset, years	Residence country
1	0.0, DBS (4–23 nmol/h/mL)	c.1340G > A	c.1552‐1G > A	NP	Speech delay	Unknown	Unknown	No	4.7	NA	NA	NA	NA	NA	NA	NA	NA	NA	Brazil
2	3.06, Saliva (31.7–251.81 nmol/mg/h)	c.1048C > T	Wild type	Unknown	Seizures	1	No others	No	1	4	3	NA	3.5	Never spoke	6	3	5	NA	Colombia
3	5.7, Leukocytes (93–521 nmol/h/ mg prot)	c.622C > T	c.622C > T	CB	Speech delay	3	Seizures, ataxia, myoclonus, pyramidal signs, cogntive decline	No	3.3	3.3	3.9	3.5	3.5	3	5	7	NA	5	Brazil
4	10.0, Leukocytes (93–521 nmol/h/ mg prot)	In analysis	In analysis	NP	Seizures	3	Cognitive decline, language difficulties	No	3	4	5	4	3	3	5	8	NA	5	Brazil
5†	0.0, DBS (4–23 nmol/h/mL)	c.1438G > A	1076‐2A > T	NP	Seizures	3	No others	Yes	3	NA	NA	NA	NA	NA	NA	NA	NA	Unkown	Colombia
6	2.8, DBS (4–23 nmol/h/mL)	c.1340G > A	c.1439 T > G	CB; GROD	Language delay	3.5	No others	No	7.5	5.5	8	8	6	3.5	7	8	8	6	Brazil
7	2.9, Leukocytes (91–323 nmol/h/ mg prot)	c.1226G > A	c.887‐10A > G	NP	Visual hallucination	3.5	No others	No	7	9	11	12	11	12	13	12	NA	NA	Argentina
8	5.0, Leukocytes (93–521 nmol/h/ mg prot)	c.1266G > C	c.622C > T	NP	Seizures	4	Ataxia, cognitive decline, language difficulties	No	4	4.9	5	6	4.2	4	5	8	7	6	Brazil
9	0.11, DBS (0.1–0.81 nmol/spot)	c.827A > T	c.827A > T	CB	Seizures	4	No others	No	4	5	6	6	5	6	7	8	NA	5	Chile
10	0.7, DBS (4–23 nmol/h/mL)	c.1076‐2A > T	c.887‐10A > G	NP	Language difficulties	4	Cognitive decline	No	7	13	13	NA	4	4	15	14	13	NA	Colombia
11	0.0, DBS (40–279 nmol/h/punch)	c.1340G > A	c.1343C > A	NP	Language difficulties	5	No others	No	7	7	19	20	7	5	19	20	20	19	Chile
12	6.45, Leukocytes (98–292 nmol/h/mg prot)	c.622C > T	c.887‐10A > G	CB; FP	Visual loss	5	Eye movement abnormalities	No	9	9	NA	NA	11	12	NA	5	NA	5	Argentina
13†	3.8, Leukocytes (54–368 nmol/h/ mg prot)	c.1048C > T	c.1603G > C	CB; FP	Behavioural abnormalities	5	No others	Yes	NA	9	9	15	NA	14	20	NA	5	NA	Argentina
14†	0.0, Leukocytes (54–368 nmol/h/ mg prot)	c.1048C > T	c.1603G > C	NP	Behavioural abnormalities	5	No others	Yes	6	NA	NA	12	12	9	NA	NA	5	NA	Argentina
15†	0.0, DBS (40–279 nmol/h/punch)	c.827A > T	c.887‐10A > G	NP	Seizures	6	Cognitive decline, language difficulties	Yes	6	7	8	NA	6	6	8	NA	NA	NA	Chile
16	1.0, DBS (4–23 nmol/h/mL)	c.1048C > T	c.1424C > T	NP	Seizures	6	Ataxia, cognitive decline, language difficulties	No	6	6	NA	8	6	6	10	NA	6	NA	Chile
17	0.07, DBS (0.1–0.81 nmol/spot)	c.887‐10A > G	c.1424C > T	CB	Seizures	6	No others	No	6	9	NA	19	7	11	21	NA	18	NA	Chile
18	0.0, DBS (40–279 nmol/h/mL)	c.1340G > A	c.827A > T	NP	Language regression	7	No others	No	21	15	NA	15	10	10	18	17	19	NA	Argentina
19	2.9, Leukocytes (91–323 nmol/h/ mg prot)	c.887‐10A > G	c.1226G > A	NP	Seizures	7	No others	No	7	NA	NA	NA	NA	10	14	12	13	NA	Argentina
20†	2.5, Leukocytes (98–292 nmol/h/ mg prot)	c.196C > T	c.887‐10A > G	CB; FP	Behavioural abnormalities	7	No others	Yes	9	12	11	15	11	11	NA	15	7	12	Argentina
21†	0.6, Leukocytes (98–292 nmol/h/ mg prot)	c.196C > T	c.887‐10A > G	CB; FP	Behavioural abnormalities	7	No others	Yes	8.5	13	12	16	11	11	NA	15	7	12	Argentina
22	0.2, DBS (4–23 nmol/h/mL)	c.887‐10A > G	In analysis	NP	Seizures	8	Ataxia, myoclonus, language difficulties, swallowing difficulty, eye movement abnormalities	No	8	8	8	NA	NA	8	8	NA	NA	8	Brazil
23	4.8, Leukocytes (91–323 nmol/h/ mg prot)	c.622C > T	c.887‐10A > G	NP	Seizures	8	Ataxia	No	8	8.5	9	NA	10	9	NA	NA	NA	NA	Argentina
24	0.1, Leukocytes (93–521 nmol/h/ mg prot)	c.1048C > T c.1438G > A	c.1483G > A	NP	Behavioural abnormalities	9	Cognitive decline	No	NA	11	NA	11	9	20	22	22	9	NA	Colombia
25	0.7, DBS (4–23 nmol/h/mL)	c.1048C > T	c.1424C > T	CB	Cognitive impairment	9	No others	No	11	12	15	NA	9	12	15	14	14	NA	Chile
26†	0.15, DBS (0.1–0.81 nmol/spot)	c.827A > T	c.887‐10A > G	CB	Ataxia	9	No others	Yes	10	9	10	11	10	10	18	11	18	19	Chile
27	0.05, DBS (0.1–0.81 nmol/spot)	NA	NA	NP	Seizures	9	No others	No	9	13	10	NA	11	13	13	13	NA	NA	Chile
28†	0.0, DBS (4–23 nmol/h/mL)	c.1438G > A	1076‐2A > T	NP	Seizures	10	Myoclonus	Yes	10	12	10	NA	NA	NA	NA	NA	NA	NA	Colombia
29†	0.0, DBS (4–23 nmol/h/mL)	c.1438G > A	1076‐2A > T	NP	Seizures	11	Cognitive decline, language difficulties, behavioural abnormalities	Yes	11	12	NA	NA	11	11	13	NA	11	NA	Colombia
30	1.9, DBS (4–23 nmol/h/mL)	c.887‐10A > G	c.887‐10A > G	NP	Cognitive impairment	41	No others	No	NA	46.5	50	47	42	47	51	NA	43.5	51	Brazil

† Patients 5‐28‐29, 13‐14, 15‐26 and 20‐21 are siblings.

CG, curvilinear bodies; DBS, dried blood spot; FP, fingerprint; GROD, granular osmiophilic deposits; NA, not applicable or not availabe; NP, not perfomed.

Forty‐one percent (12/29) of the patients with documented age of the first symptom onset experienced at least one additional symptom before their next anniversary (i.e. at the same age of the first symptom). The most frequent concurrent symptoms were cognitive decline (67%, 8/12) and language difficulties (50%, 6/12). Interestingly, the majority of the patients (27%, 8/29) who displayed more than one symptom within 12 months had seizures as the first symptom.

The symptoms that motivated parents towards a medical consultation occurred at 7 years on average (*n* = 23, min = 1, max = 46) and these were: seizures (73%, 22/30), movement disorders (i.e. motor impairment, hyperkinesia, hypokinesia, frequent falls and movement disorders) (17%, 5/30), ataxia (10%, 3/30), behavioural abnormalities (7%, 2/32), cognitive impairment (3%, 1/30), language regression (3%, 1/30) and visual impairment (3%, 1/30). Table 2 depicts the clinical findings presented by the patients enrolled in the study throughout their lifetimes as well as the age of symptom onset and percent of patients.

**Table 2:** Clinical findings and their onset (in years) in patients with atypical CLN2 from Latin America

Clinical findings	Frequency, %	Age at symptom onset, years, mean ± SD (*n*)	Age at symptom onset (min–max)
Language difficulty	100	10.68 ± 8.53 (25)	(3.00–47.00)
Cognitive impairment	93	9.30 ± 7.57 (24)	(3.00–42.00)
Cerebellar atrophy	93	11.56 ± 9.85 (22)	(3.25–52.00)
Seizures	90	7.26 ± 3.84 (26)	(1.00–21.00)
Ataxia	90	8.72 ± 3.17 (23)	(3.25–13.00)
White matter abnormalities	87	10.03 ± 9.79 (23)	(3.25–52.00)
Cerebral atrophy	79	12.51 ± 11.45 (17)	(3.25–52.00)
Swallowing difficulty	76	14.23 ± 9.93 (22)	(5.00–51.00)
Myoclonus	73	11.30 ± 9.88 (20)	(3.00–50.00)
Pyramidal signs	63	13.47 ± 9.67 (18)	(3.50–47.00)
Visual loss	62	11.47 ± 5.04 (17)	(3.00–22.00)
Dystonia/Parkinsonism	60	12.34 ± 5.31 (16)	(5.00–21.00)
Behavioural disorders	56	10.88 ± 5.33 (17)	(5.00–20.00)
Eye movement abnormalities	40	12.75 ± 13.13 (12)	(5.00–51.00)

SD, standard deviation.

Abnormal electroencephalogram was present in 60% (18/30) of the patients in the cohort. Most abnormalities (37%, 11/30) were detected using low‐frequency photostimulation (1–3 Hz range).

Different diagnostic methods were employed (Table 3). All patients (100%, 30/30) were tested for TPP1 enzyme activity. More specifically, DBS was the most used matrix to evaluate the enzymatic activity, being employed in 57% of the patients (17/30), followed by leukocytes (40%, 12/30). The saliva evaluation was performed in one patient (3%, 1/30). Reduced TPP1 enzyme activity (from leukocytes, saliva or DBS) was verified in 93% (28/30) of patients. Of note, eight patients presented null enzyme activity while two patients displayed enzymatic activity at the lower normal limit.

**Table 3:** Combination of diagnostic methods for confirmation of CLN2 in Latin American patients

Combination	Frequency	Percent
Gene panel + enzyme activity assay	7	23
Gene panel + enzyme activity assay + skin biopsy	6	20
Enzyme activity assay exclusively	6	20
Enzyme activity assay + exome	6	20
Enzyme activity assay + skin biopsy	2	7
Enzyme activity assay + genotyping	1	3
Enzyme activity assay + exome + metabolomic analysis	1	3
Enzyme activity assay + other methods (not clarified)	1	3

Gene panels for identification of genetic mutations were used in 47% of the patients (14/30); whole exome sequencing was used in 23% (7/30) and other methods (i.e. genotyping, metabolomic analysis and skin biopsy) were used in 37% of the patients (11/30). Diagnostic confirmation was achieved by both identification of genetic mutation and TPP1 enzymatic activity in 23% (7/30) of the patients, and exclusively by enzymatic activity assay in 20% (6/30) of the patients, as shown in Table 3. One patient had the CLN2 diagnosis confirmed by metabolomic analysis.

The most frequent mutations in this cohort were c.887‐10A > G (13 alleles) and c.1048C > T (6 alleles). One patient presented as homozygous for the intronic mutation c.827A > T and three novel mutations were detected in our population: c.1226G > A, c.1343C > A and c.1552‐1G > A.

In this cohort, 30% (9/30) patients have affected siblings (data detailed in Table 1). By the date of the Advisory Board Meeting (October 2018), 27% (8/30) of patients had died, at the median age of 19.5 years (min = 9, max = 60).

## Discussion

The major findings of this cohort of Latin American patients with atypical CLN2 were: (i) median age at symptom onset was 6 years; (ii) the most common first symptoms were seizures (47%), followed by language abnormalities (20%) and by behavioural abnormalities (17%); (iii) during the disease course, the most common symptoms were: language difficulty (100% of patients); cognitive impairment (93%), seizures and ataxia (90%, each) and (iv) three novel mutations were found in the studied population.

The classic phenotype of CLN2 is associated with seizures, movement disorders and ataxia.^16^, ^17^, ^18^ Language delay has been described as one of the earliest manifestations of the disease, occurring in children between 2 and 4 years of age.^16^, ^19^ In our cohort, patients initially presented with seizures and language abnormalities and these occurred nearly at the age of 6 years. Of note, seizure onset was the symptom that led the majority of the families to seek medical care, while more subtle symptoms such as behavioural or cognitive changes were not enough for the families in this study to seek medical attention in the early phases of the disease.

Early diagnosis of classic CLN2 disease still constitutes a challenge despite the development of clinical diagnostic algorithms and the availability of CLN2 genetic testing and TPP1 enzyme assays.^20^ In our study, all patients were tested for enzyme activity. Although the majority of patients presented with reduced levels of enzyme activity, two patients (#9 and #26) had their enzyme levels within the normal range but at the lower limit. Their ages of onset were 10 and 4 years and showed CB inclusions by electron microscopy in addition to the presence of the previously described disease‐causing mutation c.887‐10A > G. These findings reinforce the benefit of a combination of diagnostic methods. Gene panels for the identification of genetic mutations were the second test chosen by the experts, being performed in nearly half of the studied population. Of note, the study of metabolites can be applied as a diagnostic tool in inborn errors of metabolism when previous tests are inconclusive^21^ and some work is available regarding these technologies in Batten disease.^22^ Metabolomic analysis was carried out in one of the patients in this study who had inconclusive TPP1 activity results (at the lower limit of normal in two independent samples of DBS and below normal in saliva and leukocytes) and heterozygous pathogenic variant c.1048C > T (cln2.139) in the *TPP1* gene (the other allele is wild type). Interestingly, this patient had a very early disease onset with seizures at 1 year of age, progressing with visual loss, myoclonus and cognitive decline. Results of the metabolomic analysis of this patient will be published separately in a case report.

Complete correlation between specific mutations associated with CLN2 disease and the various phenotypes is not yet available and challenged by the large number of mutations described, the epidemiology of the disease (rare condition) and the variability in clinical presentation.^3^ The most frequent mutations present in this cohort were c.887‐10A > G (cln2.079, 13 alleles) and c.1048C > T (cln2.139, 6 alleles). The c.887‐10A > G mutation was first described in CLN2 disease in Portugal,^23^ and the authors proposed that the mutant protein might retain some residual activity, leading to a late‐onset and delayed progressive form of CLN2.

The c.1048C > T (cln2.139) mutation was described in six patients of this cohort (patients #2, #13, #14, #16, #24, #25). There is evidence that this is a disease‐associated variant, since it has been reported in homozygous form in an Argentinian patient^2^ and as a compound heterozygote in a patient reported by Williams.^24^ In one patient in this study (#2), it was the only variant allele detected. This patient had below normal level of TPP1, and extremely early onset. A second disease allele therefore remains to be detected and may be located in areas of the gene not sequenced. Another patient (#24) with this allele was also homozygous for a variant of unknown significance c.1438G > A.

Three novel mutations were detected in our population: c.1226G > A, c.1343C > A and c.1552‐1G > A; the impact of these mutations on TPP1 structure, activity and phenotype remains to be determined.

The limitations of the present study include limited sample size, the retrospective nature of data as well as limiting data retrieval in clinical charts. In this regard, for example, we could retrieve general information as ‘language abnormalities’ from the chart instead of specific details of the clinical manifestation.

## Conclusion

Atypical presentation of rare diseases such as CLN2 poses additional challenges to early diagnosis. Data from this cohort of Latin American patients with atypical CLN2 show that potential differences between atypical and classical phenotypes include a later onset of disease, and that motor manifestations may not be so common as the first symptoms. In light of the availability of specific therapy that may alter disease progression, these findings strengthen the importance of clinical suspicion and investigation of CLN2 even in patients above the classical age of the disease onset who present mainly with seizures but also language or behaviour abnormalities.

## References

[1] Setty G , Saleem R , Khan A , Hussain N . Atypical juvenile neuronal ceroid lipofuscinosis: A report of three cases. J. Pediatr. Neurosci. 2013; 8: 117–9. 24082928 10.4103/1817-1745.117840 PMC3783717

[2] Kohan R , Carabelos MN , Xin W *et al*. Neuronal ceroid lipofuscinosis type CLN2: A new rationale for the construction of phenotypic subgroups based on a survey of 25 cases in South America. Gene 2013; 516: 114–21. 23266810 10.1016/j.gene.2012.12.058 PMC3855401

[3] Elleder M , Dvoráková L , Stolnaja L *et al*. Atypical CLN2 with later onset and prolonged course: A neuropathologic study showing different sensitivity of neuronal subpopulations to TPP1 deficiency. Acta Neuropathol. 2008; 116: 119–24. 18283468 10.1007/s00401-008-0349-3 PMC2956886

[4] Fietz M , AlSayed M , Burke D *et al*. Diagnosis of neuronal ceroid lipofuscinosis type 2 (CLN2 disease): Expert recommendations for early detection and laboratory diagnosis. Mol. Genet. Metab. 2016; 119: 160–7. 27553878 10.1016/j.ymgme.2016.07.011

[5] Williams RE , Adams HR , Blohm M *et al*. Management strategies for CLN2 disease. Pediatr. Neurol. 2017; 69: 102–12. 28335910 10.1016/j.pediatrneurol.2017.01.034

[6] Nickel M , Simonati A , Jacoby D *et al*. Disease characteristics and progression in patients with late‐infantile neuronal ceroid lipofuscinosis type 2 (CLN2) disease: An observational cohort study. Lancet Child. Adolesc. Health 2018; 2: 582–90. 30119717 10.1016/S2352-4642(18)30179-2 PMC7516285

[7] Bennett MJ , Rakheja D . The neuronal ceroid‐lipofuscinoses. Dev. Disabil. Res. Rev. 2013; 17: 254–9. 23798013 10.1002/ddrr.1118

[8] Gardner E , Bailey M , Schulz A , Aristorena M , Miller N , Mole SE . Mutation update: Review of TPP1 gene variants associated with neuronal ceroid lipofuscinosis CLN2 disease. Hum. Mutat. 2019; 40: 1924–38. 31283065 10.1002/humu.23860 PMC6851559

[9] Mole SE , Anderson G , Band HA *et al*. Clinical challenges and future therapeutic approaches for neuronal ceroid lipofuscinosis. Lancet Neurol. 2019; 18: 107–16. 30470609 10.1016/S1474-4422(18)30368-5

[10] Markham A . Cerliponase alfa: First global approval. Drugs 2017; 77: 1247–9. 28589525 10.1007/s40265-017-0771-8

[11] Schulz A , Ajayi T , Specchio N *et al*. Study of intraventricular cerliponase alfa for CLN2 disease. N. Engl. J. Med. 2018; 378: 1898–907. 29688815 10.1056/NEJMoa1712649

[12] Di Giacopo R , Cianetti L , Caputo V *et al*. Protracted late infantile ceroid lipofuscinosis due to TPP1 mutations: Clinical, molecular and biochemical characterization in three sibs. J. Neurol. Sci. 2015; 356: 65–71. 26143525 10.1016/j.jns.2015.05.021

[13] Chen ZR , Liu DT , Meng H *et al*. Homozygous missense TPP1 mutation associated with mild late infantile neuronal ceroid lipofuscinosis and the genotype‐phenotype correlation. Seizure 2019; 69: 180–5. 31059981 10.1016/j.seizure.2018.08.027

[14] Dy ME , Sims KB , Friedman J . TPP1 deficiency: Rare cause of isolated childhood‐onset progressive ataxia. Neurology 2015; 85: 1259–61. 26224725 10.1212/WNL.0000000000001876

[15] Sun Y , Almomani R , Breedveld GJ *et al*. Autosomal recessive spinocerebellar ataxia 7 (SCAR7) is caused by variants in TPP1, the gene involved in classic late‐infantile neuronal ceroid lipofuscinosis 2 disease (CLN2 disease). Hum. Mutat. 2013; 34: 706–13. 23418007 10.1002/humu.22292

[16] Nickel M , Schulz A , Kohlschütter A *et al*. Late language acquisition and unexplained epilepsy are indicators of easily detectable CLN2 disease. Eur. J. Paediatr. Neurol. 2015; 19: S38.

[17] Chang M , Davidson BL , van Diggelen OP *et al*. CLN2. In: Mole SE , Williams R , Goebel HH , eds. The Neuronal Ceroid Lipofuscinoses (Batten Disease), 2nd edn. Oxford: Oxford University Press; 2011.

[18] Worgall S , Kekatpure MV , Heier L *et al*. Neurological deterioration in late infantile neuronal ceroid lipofuscinosis. Neurology 2007; 69: 521–35. 17679671 10.1212/01.wnl.0000267885.47092.40

[19] Steinfeld R , Heim P , von Gregory H *et al*. Late infantile neuronal ceroid lipofuscinosis: Quantitative description of the clinical course in patients with CLN2 mutations. Am. J. Med. Genet. 2002; 112: 347–54. 12376936 10.1002/ajmg.10660

[20] Schulz A , Kohlschütter A , Mink J , Simonati A , Williams R . NCL diseases – Clinical perspectives. Biochim. Biophys. Acta 2013; 1832: 1801–6. 23602993 10.1016/j.bbadis.2013.04.008 PMC4631127

[21] Katz ML . Decreased plasma carnitine and trimethyl‐l‐lysine levels associated with lysosomal accumulation of a trimethyl‐l‐lysine containing protein in Batten disease. Biochim. Biophys. Acta 1996; 1317: 192–8. 8988235 10.1016/s0925-4439(96)00054-3

[22] Kline RA , Wishart TM , Mills K , Heywood WE . Applying modern Omic technologies to the neuronal ceroid lipofuscinoses. Biochim. Biophys. Acta Mol. Basis Dis. 2020; 1866: 165498. 31207290 10.1016/j.bbadis.2019.06.012

[23] Bessa C , Teixeira CA , Dias A *et al*. CLN2/TPP1 deficiency: The novel mutation IVS7‐10A>G causes intron retention and is associated with a mild disease phenotype. Mol. Genet. Metab. 2008; 93: 66–73. 17959406 10.1016/j.ymgme.2007.08.124

[24] University College London . *CLN2 Mutation Table to View*. Gardner E and Mole S; 2020. Available from: https://www.ucl.ac.uk/ncl/CLN2mutationtable.htm [accessed June 2020].

